# Health Behaviour Change in Weight Management for People With Higher Weight and Symptoms of Common Mental Health Problems: A Qualitative Study

**DOI:** 10.1111/hex.70906

**Published:** 2026-10-25

**Authors:** Veronika Rysinova, Sarah Brown, Nicola Marie Stock

**Affiliations:** ^1^ School of Social Sciences University of the West of England (UWE) Bristol UK; ^2^ Thrive Tribe London UK; ^3^ Centre for Appearance Research, School of Social Sciences University of the West of England (UWE) Bristol UK

**Keywords:** behaviour change, COM‐B, common mental health problems, patient and public involvement, qualitative, Theoretical Domains Framework, weight management

## Abstract

**Background:**

Adults with higher weight experience disproportionate rates of common mental health problems (CMHPs), including depression, anxiety and post‐traumatic stress disorder. Tier 2 Weight Management Services (T2WMS) in the United Kingdom emphasise diet and physical activity with limited psychological input. Evidence on how people with co‐occurring CMHPs symptoms experience health behaviour change within these programmes remains limited.

**Objective:**

To explore how adults with higher weight and symptoms of CMHPs engage with health behaviour change in T2WMS, incorporating service user and practitioner perspectives, and to identify barriers, facilitators and service implications.

**Design:**

Qualitative study using semi‐structured interviews.

**Setting and Participants:**

Service users (*n* = 11) and healthcare professionals (*n* = 11) recruited from UK T2WMS.

**Main Variables Studied:**

Barriers and facilitators to eating behaviours, physical activity, emotion regulation and self‐regulation behaviours.

**Main Outcome Measures:**

Thematic patterns were analysed using codebook thematic analysis within a critical realist frame, with deductive mapping to the COM‐B model and Theoretical Domains Framework (TDF).

**Results:**

Five themes captured a dynamic interplay of emotional, social and structural factors: (1) The emotional weight of change, (2) Health on the margins of daily life, (3) Connection and judgement shape the journey, (4) Treating the whole person, and (5) Self‐care as a foundation for lifelong change. Barriers to health behaviour change included emotional fluctuation, stigma, low self‐efficacy, relational pressures and structural constraints. Facilitators included self‐compassion, emotional regulation, small achievable goals, inclusive groups and supportive practitioner relationships.

**Discussion and Conclusions:**

Provision centred on weight loss as the primary measure of success, delivered in a standardised format, was poorly aligned with the needs of those with symptoms of CMHPs. Embedding trauma sensitivity, relational safety, flexible pacing and broader outcome measures may better support sustained engagement. Mapping to COM‐B/TDF and Behaviour Change Wheel intervention functions offers a psychologically informed framework to guide commissioners and practitioners.

**Patient or Public Contribution:**

An advisory group of four members (two with lived experience of higher weight and symptoms of CMHPs, two healthcare professionals delivering T2WMS) contributed to study design, recruitment materials and interview guides shaping the study's non‐stigmatising terminology. All research participants were also invited to review preliminary findings, which informed refinements to thematic language. Patient and public involvement was documented using the GRIPP2 short‐form framework.

## 1 Introduction

Rates of higher body weight and common mental health problems (CMHPs) such as depression, anxiety and post‐traumatic stress disorder (PTSD) have risen over the past two decades and represent major intersecting public health challenges [1, 2]. In the United Kingdom, approximately one in five individuals aged 16–64 in England experience a common mental health condition in any given week [2], while an estimated two‐thirds of all adults are living with higher weight [3]. The relationship between these conditions is bidirectional; higher weight increases the risk of depression by 55%, while depression increases the risk of developing higher weight by 58% [4]. The mechanisms underpinning this association are complex and multidimensional, spanning inflammatory and neuroendocrine pathways, effects of psychotropic medication, weight stigma, emotional eating, and lower physical activity [4, 5, 6, 7]. Importantly, higher weight and CMHPs frequently co‐occur, reinforcing one another and reflecting broader structural environments such as food systems, urbanisation and unequal access [8]. The coexistence of higher weight and CMHPs compounds morbidity [9], reduces quality of life [10] and increases healthcare costs [11, 12], yet this intersection remains under‐recognised in routine practice, with people frequently reporting unmet psychological needs and additional barriers to accessing support [13, 14].

UK weight management is delivered through a four‐tiered system, from universal prevention (Tier 1) through structured community programmes (Tier 2), specialist multidisciplinary services (Tier 3) to bariatric surgery (Tier 4) [15]. Tier 2 Weight Management Services (T2WMS) are positioned as the ‘first line treatment' in the United Kingdom [16], typically delivered over 8–12 weeks via group sessions, individual consultations or digital platforms, combining dietary guidance, physical activity encouragement, self‐monitoring and behavioural strategies [17, 18]. Despite recent government investment expanding provision [16], significant structural and evidence gaps persist. Programmes retain a strong emphasis on weight loss as the primary measure of success and rarely integrate psychological support, even though over half of participants in some studies report insufficient mental health provision. [13, 14] Access to specialist Tier 3 support is geographically inconsistent, leaving many people with complex needs reliant on Tier 2 programmes [19].

People living with CMHPs encounter a wide range of barriers to health behaviour change. Weight stigma, both experienced and internalised, is associated with lower self‐esteem, disordered eating, avoidance of physical activity and disengagement from services [20, 21]. Cognitive and emotional load, medication side effects, chronic stress and social isolation further constrain engagement [22, 23]. Conversely, facilitators include emotional regulation skills, self‐compassion, supportive relationships, peer modelling, flexible programme design and approaches centred on personal values rather than weight alone [23, 24, 25, 26]. Much of this evidence on barriers and facilitators alike, however, derives from populations with serious mental health problems or other long‐term conditions such as type 2 diabetes, rather than CMHPs specifically, despite CMHPs being considerably more prevalent, affecting an estimated 22.6% of adults in England [2], compared with approximately 1% living with severe mental health problems [27]. In addition, most studies remain quantitative and outcome‐focused, with few qualitative accounts exploring the lived experience of co‐occurring higher weight and CMHPs symptoms within T2WMS specifically [14, 28].

This evidence gap is significant because quantitative studies, while demonstrating associations between CMHPs and poorer weight management outcomes [28, 29, 30], cannot illuminate how or why engagement breaks down in practice. Qualitative research with populations experiencing both higher weight and CMHPs is scarce [14], and studies that do exist tend to focus on either weight management or mental health in isolation rather than at their intersection. Furthermore, few studies have incorporated the perspectives of practitioners alongside those of service users, missing an opportunity to triangulate accounts of what helps, what hinders and what could be redesigned [13]. Without this depth of understanding, service commissioners and practitioners lack the evidence needed to tailor programmes to the complex reality of lived experience that many participants bring [14]. To help with this, this study draws on the COM‐B model (capability, opportunity, motivation – behaviour) [31] and the Theoretical Domains Framework (TDF) [32], which together provide a systematic behavioural diagnosis of what must change for a behaviour to occur.

This study aimed to explore how adults with higher weight and symptoms of CMHPs engage with health behaviour change in T2WMS, incorporating the perspectives of both service users and practitioners. Specifically, it sought to understand the barriers and facilitators experienced across eating, physical activity, emotion regulation and self‐regulation behaviours; to examine how these map onto COM‐B and TDF domains; and to identify implications for more inclusive, person‐centred service design.

## 2 Methods

### 2.1 Study Design and Philosophical Stance

A qualitative study using semi‐structured interviews was conducted, underpinned by a critical realist ontology and contextualist interpretivist epistemology [33, 34]. Critical realism was selected because it acknowledges that barriers and facilitators to health behaviour change exist independently of participants' accounts (e.g., financial strain, service structures), while recognising that their meaning and impact are shaped by individual perception, social context and lived experience. This positioned the analysis to move beyond surface‐level description to examine how emotional, relational and structural mechanisms interact to enable or constrain engagement with health behaviour change.

### 2.2 Theoretical Framework

The COM‐B model [31] and TDF [32] provided the theoretical foundation for the study. COM‐B proposes that behaviour arises from the interaction of Capability (psychological and physical), Opportunity (social and physical) and Motivation (reflective and automatic), while TDF elaborates these components into 14 domains that enable more granular behavioural diagnosis [32]. Together, they offer a systematic approach to identifying what needs to change for a behaviour to occur and what intervention strategies are most likely to be effective [35]. The Behaviour Change Wheel (BCW), within which COM‐B sits, extends this diagnostic approach by linking identified targets to intervention functions and policy categories, providing a route from behavioural analysis to practical recommendations [31]. COM‐B has been widely applied in health psychology [35], with recent work extending its use to clusters of interrelated behaviours operating simultaneously [36], consistent with the multi‐behaviour focus of this study. Unlike most COM‐B applications, which focus on a single target behaviour, this study applied the framework to four interrelated behaviour domains: eating, physical activity, emotion regulation and self‐regulation, reflecting the reality that T2WMS expect participants to change multiple behaviours simultaneously. COM‐B and TDF were used in three interconnected ways: to inform the interview topic guide by mapping questions to TDF domains while remaining open to inductive exploration; to guide the deductive mapping of inductively generated codes and themes back to COM‐B and TDF; and to shape practical implications through linkage to BCW intervention functions [35].

### 2.3 Setting and Participants

Participants were recruited from UK T2WMS over approximately 6 months (October 2024 to March 2025) through programme providers, social media and community outreach, using purposive and convenience sampling. Recruitment for lived‐experience participants was primarily via referrals from staff at partner private T2WMS clinics, supplemented by posters, leaflets and targeted posts on social media (Facebook, LinkedIn, X); because early uptake through these routes was low, a GDPR‐compliant text invitation was subsequently sent to 425 individuals identified through clinic‐held mailing lists, which yielded the majority of the final lived‐experience sample. HCPs were recruited by the first author (V.R.) directly through T2WMS team meetings, clinic leaflets and internal email circulations, supplemented by snowball sampling. Participants did not receive reimbursement. Two groups were recruited: healthcare professionals (HCPs) delivering T2WMS within the past 3 years, and individuals with higher weight and self‐reported symptoms of CMHPs [37] who had participated in T2WMS within the same period. Programme completion was not required for eligibility. Sample adequacy was assessed using the concept of information power [38]. This was assessed via sample specificity to the research question (higher weight, CMHP, T2WMS, dual perspectives), use of an established theoretical framework (COM‐B, TDF), dialogue quality during interviews (mean = 56 min) and a cross‐case thematic analytic strategy. Ethical approval was obtained from the University of the West of England Faculty Research Ethics Committee (REF No: CHSS.24.05.201).

The final sample comprised 22 participants: 11 individuals with lived experience (age range 34–76 years [mean = 48 ± 13.9]; 10 identifying as female and one male; predominantly White ethnicity) and 11 HCPs (age range 23–62 years [mean = 38 ± 12.6]; 9 identifying as female and two males; predominantly White ethnicity). Lived experience participants reported symptoms of depression (*n* = 9), generalised anxiety (*n* = 7), panic symptoms (*n* = 3), OCD symptoms (*n* = 2) and PTSD‐related symptoms (*n* = 2), with most reporting multiple CMHPs symptoms. Among lived‐experience participants, programme engagement ranged from 2 to 4 weeks (1) to > 12 weeks (6) and one long‐term member. Among HCPs experience delivering programme ranged from less than 1 year (3) to over 5 years (2). Both groups engaged with a range of delivery formats including one‐to‐one, group, online and face‐to‐face.

### 2.4 Patient and Public Involvement and Engagement

Patient and public involvement and engagement (PPIE) was embedded throughout the research process. An advisory group (*n* = 4; two individuals with lived experience of higher weight and CMHPs symptoms, two HCPs delivering T2WMS) contributed to study design, ethics application, participant‐facing materials and the interview topic guide, ensuring language was accessible, non‐stigmatising and relevant to the experiences of both groups. Members were identified through existing service networks and professional contacts and invited by email or verbally. Three members took part in individual single virtual meetings (30–60 min) with VR, covering the clarity and tone of participant materials, the acceptability of recruitment processes and the sensitivity of proposed interview questions; one member preferred to contribute written feedback by email. Meeting members individually was intended to reduce the potential for HCP members' professional standing to influence lived‐experience members' contributions. Feedback was incorporated iteratively into study design and materials, with final decisions resting with the research team. Advisory group members were not remunerated for their contributions. Following each meeting, VR emailed members to confirm which suggestions had been implemented and to invite further feedback and no further feedback was raised.

One advisory member also took part as an interview participant; their advisory and interview contributions were treated as distinct inputs and their advisory contribution was given before their interview took place. The advisory group's input produced tangible changes, including recruitment materials being reworded for accessibility (e.g. ‘take part’ rather than ‘participate,’ ‘people’ rather than ‘individuals’), a line in the participant information leaflet that could be misread being clarified, and the topic guide was expanded to address emotional readiness. The terms ‘common mental health problems’ (rather than ‘disorders’ or ‘conditions’) and ‘higher weight’ (rather than ‘obesity’ or ‘overweight’) were adopted following advisory‐group feedback, to reflect non‐stigmatising, public‐facing language. Involvement extended into the analytic phase through participant review of preliminary findings (see Section 2.6). PPIE activities were documented using the GRIPP2 short‐form framework [39] (see File S1).

### 2.5 Data Collection

Eligible participants completed an online eligibility screening and demographic questionnaire via Qualtrics prior to interview. Prospective participants who responded to study recruitment materials (clinic referral, poster/social media advertisement, or text invitation) were directed to an online eligibility screener via a link included in the relevant recruitment material or provided by the referring clinic staff member. A brief initial question asked whether respondents had taken part in a T2WMS programme within the past 3 years and had experience of CMHPs; those responding yes proceeded to the information sheet and consent form. Eligibility for the lived‐experience group required self‐reported current or past experience of CMHPs, assessed via a multi‐select item asking participants to indicate which of several symptom clusters (depressive, generalised anxiety, panic, obsessive‐compulsive, and post‐traumatic stress symptoms), described using NICE‐informed symptom descriptors [40], applied to them; no formal diagnosis or validated cut‐off score was required. Severe cognitive difficulties or severe mental health problems were exclusion criteria for this study; however, no respondents met this exclusion criterion.

Semi‐structured interviews were then conducted via Microsoft Teams by VR, who had structured training in qualitative methods and prior experience conducting interviews in health contexts. Interviews ranged from 38 to 68 min (mean = 56 min). Separate but comparable schedules were developed for lived experience participants and HCPs to ensure contextual relevance (see File S2). All interviews were audio‐recorded with consent, transcribed verbatim and managed using NVivo 14 [41]. Participants were assigned pseudonyms to protect their anonymity, and no non‐participants were present during interviews.

### 2.6 Data Analysis

Data were analysed using codebook thematic analysis [42], selected for its structured yet flexible approach suited to applied health research. Analysis followed Braun and Clarke's six‐phase framework including familiarisation with the data, initial coding, generating themes, reviewing themes, defining and naming themes, and producing the report [43]. Inductive, line‐by‐line coding was conducted by VR primarily at a semantic level, with some latent interpretation, generating a living codebook with definitions and illustrative examples for each code. Codes from lived experience and HCP data were initially developed separately to preserve the distinctiveness of each perspective before being compared and integrated. Where lived experience and HCP accounts diverged, both perspectives were retained within the relevant theme rather than merged into a single narrative, preserving areas of convergence, divergence and conflict identified during integration (see Section Design, 3). In a planned deductive step, inductively generated themes were mapped to COM‐B and TDF domains to ensure coverage of behavioural drivers and to clarify proposed mechanisms of action [32, 35]. Trustworthiness was enhanced through a detailed coding manual, audit trail, regular monthly supervisory review, and the inclusion of diverse participant quotations across both groups. Supervisory review by SB and NMS covered the codebook and thematic outputs at multiple stages (initial themes and subthemes, subsequent revisions, and the final thematic structure) and a sample of raw coded transcripts. Mapping was conducted at the theme level following initial inductive coding. VR assigned each theme and subtheme to the COM‐B component(s) and TDF domain(s) it most directly reflected, with outputs reviewed by the supervisory team (S.B. and N.M.S.). The mapping was further refined following the doctoral viva examination, informed by feedback from an examiner with expertise in the COM‐B model.

VR had professional experience in weight management services, which informed sensitivity to participants' accounts and facilitated rapport, but also required ongoing reflexive examination of assumptions through journaling and supervision. This background may have positioned the researcher as something of an ‘insider’ in relation to HCP participants, sharing professional language and context, and more of an ‘outsider’ in relation to lived‐experience participants. This asymmetry may have also shaped rapport and interpretation differently across the two groups. For example, it may have facilitated rapport with HCPs, oriented attention toward service‐level rather than individual‐level explanations and required deliberate effort to build trust and minimise perceived power differentials with lived‐experience participants, who may have anticipated judgement given the researcher's clinical background.

Reporting followed the Consolidated Criteria for Reporting Qualitative Research (COREQ, see File S3) [44]. As a check on interpretive validity, all participants were invited to review preliminary thematic summaries; feedback from 11 (50%) refined thematic language and emphasis. For example, clarifying that ‘weight loss alone was rarely enough’ referred specifically to sustaining motivation, and making the perceived value of T2WMS more explicit. This provided a check on researcher assumptions and strengthened the trustworthiness of the themes.

## 3 Results

Five overarching themes and 13 subthemes were identified, capturing the emotional, social and structural factors shaping engagement with health behaviour change (see Table 1 for key themes, subthemes and COM‐B/TDF mapping and Figure 1 for the thematic map).

**Table 1:** Themes, subthemes and COM‐B/TDF mapping.

Theme	Subtheme	COM‐B domains	Key TDF domains
1. The emotional weight of change	1.1 When mood disrupts momentum	Psychological capability; Reflective and automatic motivation; Social opportunity	Emotion; Beliefs about capabilities; Beliefs about consequences, Behavioural regulation; Social influences; Social/professional role and identity; Reinforcement, Memory, attention and decision processes
	1.2 How stigma shapes self‐worth		
	1.3 Defined by the numbers		
	1.4 Stuck between knowing and doing		
2. Health on the margins of daily life	2.1 When life leaves little room for health	Physical and social opportunity; Psychological capability; Reflective and automatic motivation	Emotion, Environmental context and resources; Social influences; Behavioural regulation; Beliefs about capabilities; Knowledge; Skills
	2.2 Support that fits into realities of life		
3. Connection and judgement shape the journey	3.1 Feeling judged, compared or misunderstood	Social opportunity; Reflective and automatic motivation	Social influences; Emotion; Beliefs about capabilities, Social/professional role and identity
	3.2 Finding connection, belonging and trust		
4. Treating the whole person	4.1 Mind and body go hand in hand	Psychological and Physical capability; Reflective and Automatic motivation; Physical and social opportunity	Knowledge; Skills; Emotion; Beliefs about capabilities; Beliefs about consequences, Behavioural regulation; Reinforcement; Goals, Environmental context and resources
	4.2 Movement and small steps as emotional regulation		
	4.3 Reframing progress with compassion		
5. Self‐care as a foundation for lifelong change	5.1 Anchoring change in meaning and values	Reflective and Automatic motivation; Psychological capability; Physical and social opportunity	Emotion; Beliefs about consequences; Social/professional role and identity; Goals; Intentions; Behavioural regulation; Optimism
	5.2 Living change as a lifelong journey		

**Figure 1:**
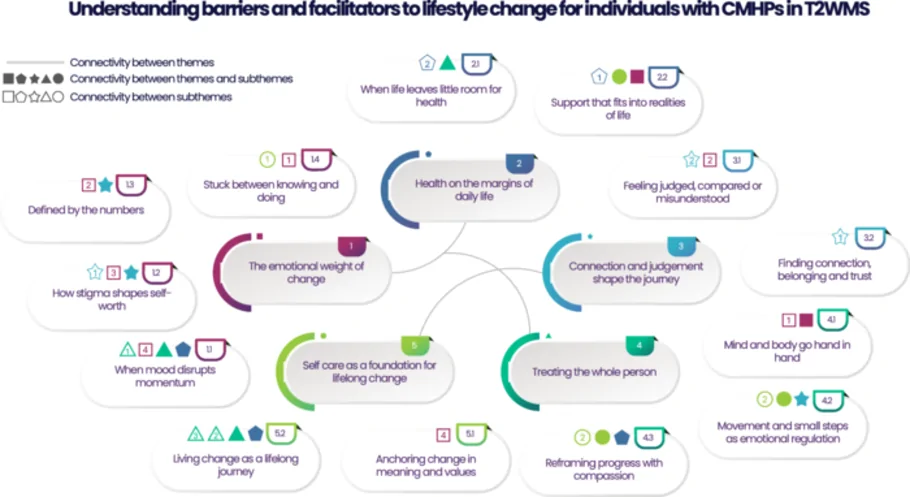
Thematic map illustrating five overarching themes and 13 subthemes, with interconnections between emotional, social and structural factors shaping engagement with health behaviour change in T2WMS.

Throughout, quotations are attributed by pseudonym and participant group (lived experience or HCP). Where an HCP quote recounts a client's reported experience rather than the practitioner's own, this is noted in the attribution (e.g., recounting a client's account). To preserve anonymity in this small sample, no further individual‐level demographic detail is reported beyond the aggregate summary in Table 2.

**Table 2:** Summary of participant characteristics.

Characteristic	Lived experience (*n* = 11)	HCPs (*n* = 11)
Age, years: range (mean)	34–76 (48)	23–62 (38)
Gender: female/male	10/1	9/2
Ethnicity		
White	9	10
Asian/Asian British	1	1
Mixed/Multiple	1	—
Education		
GCSEs	1	—
A levels or equivalent	3	2
Undergraduate qualification	2	5
Postgraduate qualification	5	4
Employment		
Full‐time	6	8
Part‐time	2	3
Self‐employed	1	—
Retired	1	—
Carer	1	—
CMHPs symptoms self‐reported/observed in clients		
Depression	9	11
Generalised anxiety	7	10
Panic symptoms	3	5
OCD symptoms	2	6
PTSD‐related	2	5
Delivery format		
Group	7	6
One‐to‐one	4	6
Online	5	6
Face‐to‐face	8	7
Engagement duration/delivery experience		
2–4 weeks	1	< 1 year: 3
4–6 weeks	1	1–2 years: 2
6–12 weeks	2	2–3 years: 1
> 12 weeks^a^	6	3–5 years: 3
Long‐term member^a^	1	> 5 years: 2

a In the United Kingdom, T2WMS commissioning typically funds a maximum of 12 weeks' access per enrolment per year, participants coded as ‘> 12 weeks’ or ‘long‐term member’ therefore reflect either repeated annual re‐enrolment or, for some programmes, continued attendance via self‐funded or programme‐extended membership beyond the commissioned period.

### 3.1 Theme 1: The Emotional Weight of Change

Efforts to change health behaviours were closely entangled with mental health. Both groups emphasised the heavy emotional and cognitive load involved, rather than any lack of knowledge. Fluctuating mood, anxiety and fatigue disrupted routines and produced cycles of progress and withdrawal. As one participant explained: ‘Most of the time, I feel like just lying down, pulling the blankets over my head, and sleeping. I'm permanently exhausted’ (Rosa, lived experience). Others highlighted the added strain of managing mental health alongside programme demands: ‘Some weeks it felt overwhelming to keep up with it. But again, I think that was more to do with my mental health—just feeling overwhelmed in general’ (Amelia, lived experience). Practitioners observed similar patterns: ‘I lose a lot of clients by kind of week 4, week 5, because actually they don't want to leave their house anymore’ (Naomi, HCP). Both groups noted a heightened sensitivity to setbacks: ‘They feel frustrated sometimes. Like, ‘Why can't I just do this? No, not every day is a normal day’’ (Grace, HCP, recounting a pattern from client interactions).

Internalised weight stigma and shame persistently undercut confidence and motivation, often stemming from longstanding social experiences and deeply embedded self‐schemas. These narratives centred on shame, feelings of inadequacy, and perceptions of failure that undercut confidence and motivation. Stigma undermined reflective motivation and resulted in avoidance of activities like group‐based physical activity or structured weight management meetings: ‘I can't face going to any of these weight watcher type things where you have to be looked at. So I've really tried to do it on my own—and failed miserably’ (Elena, lived experience). Practitioners observed the pervasive language of failure: ‘I don't think I've delivered a single programme where the word ‘fail’ or ‘failure’ doesn't come up in that initial discussion’ (David, HCP). Low self‐worth was often expressed explicitly: ‘Thoughts and feelings of ‘Well, I'm not necessarily worthy of losing weight’… maybe it would be better if I just carried on how I was’ (Amelia, lived experience).

Repeated dieting histories tied self‐worth to numerical outcomes, creating cycles of anticipation and disappointment. Weighing was rarely experienced as neutral: ‘When you think you've had a really good week, and then you get on the scales and you haven't lost weight… that is the biggest demotivator’ (Phoebe, lived experience). Group weigh‐ins compounded this harm: ‘You get weighed and then [the group facilitator] says out loud, “What did you do wrong?”’ (Harriet, lived experience). Participants described a persistent gap between intention and action. Many participants knew what would help, preparing meals in advance, pacing physical activity, using diaries or apps, and reducing emotional eating triggers, but struggled to implement these behaviours. Mental load, low mood, anxiety and cognitive fatigue interrupted the formation of automatic routines, leading to inconsistent follow‐through. Alice (HCP) observed: ‘If your mood's low… it's not like taking care of yourself is top of your list—if it's even on your list at all.’ Bea (lived experience) captured the underlying feeling many faced: ‘We know all these things are good for us—But we still don't do them… What is it about human nature that sabotages us?’

Across this theme, HCP and lived‐experience accounts seem to converge closely. HCPs' observations of disengagement, avoidance and self‐critical language corroborated participants' own reports of emotional and cognitive load.

### 3.2 Theme 2: Health on the Margins of Daily Life

Health behaviours were often deprioritised due to financial pressures, caring responsibilities, work demands and limited infrastructure. These barriers drained psychological bandwidth and constrained access to health‐supporting resources: ‘I have a house to upkeep, I have a job to do… my mum is on her own, so I do quite a lot with her’ (Phoebe, lived experience). Financial strain made healthy eating difficult: ‘Try finding a piece of broccoli for under a quid’ (Rosa, lived experience). Environmental factors further limited physical activity: ‘Buses here are shocking. Having that really poor public infrastructure adds to it as well’ (Tessa, lived experience). Practitioners stressed that even well‐designed programmes struggle when basic needs go unmet: ‘They don't have the headspace to make a change because… it's full of other stuff’ (Beth, HCP). Life events could derail progress entirely: ‘[A client] lost her job… then… a car accident and she had concussion… people could go through some really significant issues during the programme’ (Sophie, HCP, recounting a client's account).

Both groups agreed that short, rigid programmes were insufficient. Participants described the abrupt ending of support as destabilising: ‘At the end of the programme, I was a little bit worried about how I would keep going on without that… figuring out how I could continue with that accountability’ (Amelia, lived experience). Practitioners echoed this: ‘A 12‐week programme is just—these people need months of this type of support… they aren't accountable to anybody afterwards’ (Beth, HCP). Participants called for flexible scheduling, digital access, ongoing check‐ins and annual refreshers. Practical tools including apps, planners and food‐mood diaries supported self‐monitoring and routine building: ‘In your app you can put in what you eat each day… that's been quite helpful… holding myself accountable’ (Ruth, lived experience). Yet tools were most effective when paired with human connection: ‘You've done 12 weeks with this programme… but I didn't go back to it. I'm not accountable to an app’ (Tessa, lived experience).

HCP and lived‐experience accounts converged on both the structural nature of these barriers and the inadequacy of fixed‐term support, with HCPs echoing participants' calls for longer, more flexible programme structures.

### 3.3 Theme 3: Connection and Judgement Shape the Journey

Social scrutiny, cultural expectations and comparison undermined confidence, heightened anxiety and reinforced low self‐worth, particularly for those already managing symptoms of CMHPs. Participants described concealing their programme attendance or medication use: ‘I didn't really tell many of my friends while I was doing [the programme]… I didn't feel like I had anyone I could have that discussion with’ (Amelia, lived experience). Judgement often came masked as humour or concern: ‘You used to have such lovely jewellery. You've got fat now’ (Beth, HCP, quoting a client's experience). Cultural and gendered expectations added further layers: ‘If you don't finish your plate, it's disrespectful. If you don't have seconds, it's disrespectful’ (Beth, HCP, recounting a pattern from client interactions). Comparison was reported as a source of distress: ‘There's a whole group of my friends that have gone out and got these injections and lost the weight like this… it's hard to see’ (Phoebe, lived experience).

Conversely, supportive practitioner relationships and inclusive group environments provided emotional safety that was experienced as a precondition for engagement: ‘That opportunity to just talk to someone and have someone interested in what I had to say… someone on my side’ (Bea, lived experience). Group belonging normalised setbacks and bolstered self‐belief: ‘The more they feel connected as a group, the better success you see’ (Diana, HCP). For participants facing mental health problems and low confidence, connection often became the catalyst for re‐engagement and hope.

Accounts converged in identifying social judgement and connection as central to engagement; however, HCP quotations relating to judgement were largely second‐hand reports of comments from others rather than reflections on other/own HCPs' conduct.

### 3.4 Theme 4: Treating the Whole Person

Participants recognised that mental and physical health are inseparable, and desired care reflecting the whole person. ‘You can't lose weight if your head's in the wrong place… and you can't get your head in the right place if your body isn't in the right place’ (Rosa, lived experience). Practitioners reinforced this: ‘It's not about calories in and calories out—that's a tiny piece of the puzzle’ (Sophie, HCP). Yet services often fell short: ‘Mental health is just—it's massive in a Tier 2 weight management course… a lot of people that go in to deliver this underestimate how much support they [service users] really do need’ (Naomi, HCP).

Physical movement and small, achievable steps served as emotional regulation tools, reducing distress and building confidence. ‘Afterwards… I'm really buzzing… like, ‘Yeah, let's get today!’’ (Harriet, lived experience). For participants with anxiety, gradual approaches were essential: ‘If I get over‐exerted, my brain can't seem to distinguish between healthy adrenaline and panic… so I've just had to be a bit careful with cardio’ (Edward, lived experience). Practitioners affirmed that even modest activity shifted mood: ‘Even five minutes… helped her feel she'd achieved something’ (Eve, HCP, recounting a client's account). Incremental steps extended beyond movement: ‘Just making those little changes can have such a big impact… Doing the small changes all the time has made it really, really easy for me’ (Ruth, lived experience).

Participants shifted from harsh self‐criticism toward self‐compassion and non‐scale victories, including improved energy, sleep, mood and self‐worth, which were experienced as more motivating than weight outcomes. ‘I think it really reinforced being more kind to myself. That was a big takeaway for me’ (Amelia, lived experience). Others celebrated functional gains: ‘For me, the biggest one was my energy levels and how much easier walking became… my fibromyalgia symptoms reduced significantly’ (Amelia, lived experience). Practitioners supported this reframing: ‘Climb the stairs… get in and out of the car more easily… those key reasons are linked to quality of life’ (Jade, HCP).

HCP and lived‐experience accounts converged on the general principle that mental and physical health are inseparable, with HCPs' acknowledgement that services often underestimate psychological need corroborating participants' own experiences of unmet support. However, lived‐experience participants more often described this connection as a personal realisation gained through the programme itself, a shift in understanding not as commonly reflected in HCP accounts. This was most evident among participants with longer programme engagement.

### 3.5 Theme 5: Self‐Care as a Foundation for Lifelong Change

Participants grounded behaviour change in personally meaningful values, including family, health legacy and identity. Connecting change to a ‘powerful why’ made motivation more resilient: ‘I want [my daughter] to have a good relationship with eating… Me being a better me for her’ (Ruth, lived experience). Others linked their efforts to family health histories: ‘We lost my dad to the same thing… I don't want none of that. I've got 10 grandchildren… I want to be able to see them grow up’ (Elena, lived experience). Some framed change as reclaiming identity: ‘I didn't feel like me anymore’ (Phoebe, lived experience). Practitioners emphasised the importance of uncovering this deeper purpose: ‘Finding their ‘why’ is the most important’ (Alice, HCP). ‘The bigger the reason to change, the more likely we are to change’ (David, HCP). For those living with symptoms of CMHPs, these values helped shift motivation from external pressure to internalised, identity‐based reasons for change.

Over time, participants reframed self‐care as a legitimate, lifelong practice rather than a short‐term project. ‘My journey isn't a week. My journey is now till whenever I'm not here’ (Imani, lived experience). ‘Slow and steady… can be a lifetime of good. I won't revert back’ (Ruth, lived experience). Letting go of guilt was central to this shift: ‘It was to do with self‐worth and self‐care… standing in your own corner’ (Bea, lived experience). ‘I will not feel guilty about eating any food. That's why I find a way to balance this lifestyle’ (Nisha, lived experience). Practitioners reinforced this flexible approach: ‘You can eat a chocolate bar every day. If that's important to you, let's factor it in… life's miserable without chocolate’ (Grace, HCP). ‘You're not being selfish by doing this—spending time on yourself is important’ (Beth, HCP). Importantly, participants acknowledged this was an ongoing process: ‘The sustaining's quite hard for me… I'm still practising… I don't beat myself up so much’ (Elena, lived experience). This reframing was particularly important for participants with histories of ‘yo‐yo’ dieting and self‐blame, enabling a shift from all‐or‐nothing thinking toward flexible, identity‐based motivation.

Accounts converged strongly on the centrality of personal values and self‐compassion to sustained motivation, with HCPs' own coaching language mirroring the shift toward flexible, identity‐based reasoning described by lived‐experience participants.

### 3.6 COM‐B and TDF Mapping

Mapping revealed a complex interplay across all COM‐B components. Table 3 summarises the key barriers and facilitators identified within each COM‐B domain, alongside the corresponding TDF domains. Psychological capability was recurrently constrained by fluctuating mood, cognitive fatigue and difficulty building automaticity in new routines. Physical opportunity was limited by financial strain, rigid scheduling and environmental barriers. Social opportunity operated bidirectionally. Judgement and cultural pressures undermined engagement, while empathetic relationships and inclusive groups enabled it. Both reflective motivation (shaped by beliefs about capabilities, consequences and goals) and automatic motivation (shaped by emotional associations and reinforcement) were central. Critically, capability, opportunity and motivation interacted dynamically. Emotional distress reduced perceived capability and motivation, which in turn made available opportunities harder to access, creating self‐reinforcing cycles of disengagement.

**Table 3:** COM‐B and TDF mapping of key barriers and facilitators.

COM‐B domain	Key TDF domains	Key barriers	Key facilitators
Psychological capability	Behavioural regulation; Knowledge; Skills; Memory, attention and decision processes	Cognitive fatigue and low mood disrupting self‐monitoring and planning; difficulty building automaticity; intention–action gap; overwhelming programme content	Practical tools (apps, planners, food‐mood diaries); small achievable steps and incremental goal‐setting; structured routines and cues; emotion regulation skills
Physical capability	Physical skills	Pain, fatigue, chronic conditions like fibromyalgia; panic symptoms mimicking over‐exertion	Gentle pacing, graded exposure, practitioner reassurance; adaptations for chronic conditions
Physical opportunity	Environmental context and resources	Financial strain limiting access to healthy food and activities; poor transport and infrastructure; rigid programme scheduling; abrupt programme ending with no follow‐up	Hybrid delivery (online and face‐to‐face); flexible scheduling; subsidised access; digital resources; booster sessions and maintenance contacts
Social opportunity	Social influences	Social scrutiny and comparison; cultural and gendered food expectations; family/peer judgement; concealment of programme attendance; isolation	Supportive practitioner relationships; inclusive, psychologically safe groups; peer normalisation of setbacks; group belonging and accountability
Reflective motivation	Beliefs about capabilities; Beliefs about consequences; Goals; Intentions; Social/professional role and identity	Low self‐efficacy and self‐worth; internalised weight stigma; reliance on weight‐focused metrics that tie self‐worth to numbers; repeated dieting failure; all‐or‐nothing thinking	Values‐based motivation (‘powerful why’); identity‐based reframing of self‐care; non‐scale victories (energy, mood, sleep, self‐worth); compassionate goal‐setting
Automatic motivation	Emotion; Reinforcement	Emotional eating as coping mechanism; anxiety and shame triggering avoidance; negative emotional associations with exercise and group settings; weigh‐in distress	Self‐compassion replacing self‐criticism; emotional uplift from small wins; movement as emotional regulation; positive reinforcement from practitioners and peers; reframing setbacks as part of the journey

## 4 Discussion

This study provides an in‐depth qualitative exploration of how individuals with higher weight and symptoms of CMHPs experience health behaviour change within UK T2WMS, integrating perspectives from both service users and practitioners. Five themes revealed that engagement may be shaped by a dynamic interplay of emotional, relational and structural factors. Participants discussed that fluctuating mood, anxiety and fatigue disrupted capability to engage with health behaviour change on a daily basis; internalised stigma and weight‐focused metrics eroded motivation and self‐worth; financial strain, caregiving demands and rigid programme structures constrained opportunity; social judgement drove concealment and withdrawal while psychological safety and peer connection enabled re‐engagement; and participants who anchored change in personal values and self‐compassion sustained motivation more effectively than those pursuing weight loss alone. These findings indicate that the standardised, time‐limited model that treats weight loss as the primary measure of success, underpinning much of current T2WMS provision may not, for many participants in this study, adequately accommodate the emotional variability, relational needs and structural realities of people living with co‐occurring higher weight and symptoms of CMHPs.

Consistent with prior research [5, 22, 23], psychological distress, including depression, anxiety, fatigue and emotional dysregulation, was a dominant barrier. A key novel contribution was the finding that participants' psychological capability fluctuated dynamically on a daily or weekly basis, heavily influenced by mood, stress and life circumstances. This adds important nuance to the COM‐B model [31], helping explain why individuals may disengage despite initial motivation, which is a pattern that can be often missed in single time‐point assessments. These findings support a more systems‐aware understanding in which COM‐B components interact through feedback loops rather than operating independently. This interpretation draws on West and Michie's updated formulation of COM‐B [45], in which capability and opportunity act as ‘gates’ that must be open for motivation to translate into behaviour, and behaviour is understood as arising from competition between alternative possible actions rather than a single fixed pathway.

The feedback loop between COM‐B components was particularly evident in the data. Emotional distress (automatic motivation) not only reduced perceived capability to plan and self‐monitor (psychological capability) but also narrowed perceptions of available opportunity, making even accessible resources feel out of reach. Reduced capability and narrowed opportunity in turn further diminished motivation, creating self‐reinforcing cycles of disengagement. Conversely, when participants experienced small wins, a manageable walk, a week of consistent meal planning, positive feedback from a practitioner, this created upward spirals: improved automatic motivation (emotional uplift, reinforcement) restored a sense of capability, which in turn made available opportunities feel more accessible. This dynamic, bidirectional interaction suggests that interventions targeting any single COM‐B component in isolation may be less likely to produce sustained change [45]. Instead, services may need to address the system of interactions, particularly by stabilising emotional states and building psychological safety as foundations from which capability and opportunity can be accessed.

Another central finding was the role of psychological safety as a prerequisite for meaningful engagement, particularly in group settings. Previous literature has highlighted the value of supportive relationships and trauma‐informed approaches [46, 47], but this study demonstrated that the emotional climate of safety, not merely the presence of others, was experienced as a precondition for participation among individuals with symptoms of CMHPs. Participants frequently described avoidance or withdrawal from group interventions due to fear of judgement, social comparison or reactivation of past trauma. For those who found safety within their groups, the experience was often described as transformative, providing belonging, accountability and emotional containment that facilitated re‐engagement with behaviours they had previously abandoned.

Non‐scale victories, including improved sleep, mood, energy and self‐worth, were reported to be perceived as more motivating than weight outcomes, supporting strengths‐based and self‐compassion‐oriented approaches [25, 48, 49]. Self‐compassion appeared to function not only as a protective coping strategy but also as an active driver of behaviour change, helping participants interrupt cycles of shame and avoidance and re‐engage after setbacks. This extends existing literature on the role of self‐compassion in weight management [48, 50, 51].

Structural barriers including financial strain, caregiving demands, rigid scheduling and limited follow‐up, were pervasive, echoing well‐established evidence on social determinants of health [52, 53]. The fixed 12‐week programme format was perceived as insufficient by many participants in this study, with some describing feeling ‘cut off’ when support ended. This reinforces critiques of standardised, time‐limited interventions that fail to accommodate fluctuating emotional states and the chronic, relapsing nature of behaviour change [19, 54]. Some participants reported that they valued ongoing, emotionally responsive support, such as booster sessions, peer‐based groups, emotional check‐ins, which are elements rarely included in standard service models.

### 4.1 Implications for Practice and Policy

Mapping barriers and facilitators to COM‐B/TDF domains and corresponding BCW intervention functions yields several potential actionable recommendations. First, programmes may embed structured psychological support, including content on emotional regulation, self‐compassion and coping with setbacks, within psychologically safe, stigma‐reducing environments. Co‐located or clearly signposted mental health pathways (e.g., NHS Talking Therapies for Anxiety and Depression [formerly IAPT], social prescribing) should be routinely available. Second, services may shift from evaluation focused primarily on weight to broader outcome metrics incorporating mental wellbeing, energy, sleep, functioning and quality of life alongside participant‐selected weight goals, recognising that some participants may respond better to these broader measures. This is consistent with evidence that weight stigma and its internalisation can predict health outcomes more strongly than BMI itself, highlighting the potential value of outcome measures that do not rely on weight [21, 55, 56]. Third, programme design may move from fixed‐term courses to longitudinal pathways combining an intensive entry phase with structured maintenance at 6, 12 and 24 months, using hybrid delivery (e.g., online, app‐based, in person) and adaptable pacing. Fourth, relational facilitation may be prioritised through training in trauma‐informed, culturally responsive and compassionate communication. Finally, these individual‐level recommendations may be complemented by structural action, including subsidised access, improved digital infrastructure, cross‐sector partnerships and policy measures addressing the food environment, poverty and health inequalities. Table 4 summarises key implications linked to the behavioural framework.

**Table 4:** Selected COM‐B/TDF to BCW‐mapped implications for T2WMS*.* ^a^

COM‐B domain	Key TDF domains	BCW intervention functions	Practical implications
Automatic motivation	Emotion; Reinforcement	Persuasion; Modelling; Enablement	Embed emotional regulation and self‐compassion content; create psychologically safe, stigma‐reducing environments; co‐locate mental health pathways (e.g., NHS Talking Therapies, social prescribing)
Reflective motivation	Beliefs about capabilities; Goals; Beliefs about consequences	Education; Persuasion; Modelling; Enablement	Shift from weight loss as the primary measure of success to broader outcome metrics (wellbeing, energy, sleep, functioning); use compassionate, values‐aligned goal‐setting; integrate peer modelling
Social opportunity	Social influences	Modelling; Environmental restructuring; Enablement	Build inclusive, culturally sensitive groups; sustain peer networks; train in trauma‐informed, empathetic facilitation; support role renegotiation in family/peer contexts
Physical opportunity	Environmental context and resources	Environmental restructuring; Enablement	Offer hybrid delivery and flexible scheduling; subsidise access; signpost financial/housing/transport support; improve digital infrastructure
Psychological capability	Behavioural regulation; Knowledge; Skills, Memory, attention and decision processes	Training; Education; Enablement	Embed automaticity‐building (cues, reminders); integrate booster sessions and maintenance contacts at 6, 12 and 24 months; teach emotion regulation, pacing and adaptive coping; coach self‐monitoring

^a^ The mapping reflects the authors’ interpretive linkage of study findings to BCW intervention functions.

### 4.2 Strengths and Limitations

This study has several strengths. The in‐depth qualitative design captured rich, person‐centred accounts of a population whose experiences are rarely foregrounded in weight management research. Including both service user and practitioner perspectives enabled comparative insights and strengthened the credibility of findings where accounts converged. The systematic use of codebook thematic analysis with deductive COM‐B/TDF mapping provided a structured link between lived experience and behavioural theory, and the integration of lived experience and practitioner perspectives across design, data collection and interpretation strengthened the relevance and interpretive validity of the findings.

Several limitations should be acknowledged. The sample was predominantly White and female, with limited representation of male participants, ethnically diverse communities and people who had disengaged from services, which may constrain transferability to broader populations. Recruitment via programme providers and community outreach may also have introduced self‐selection (participants with particularly salient positive or negative views opting in) and provider gatekeeping (staff may have preferentially referred more engaged clients). Eligibility did not require programme completion, and the sample did include some participants with brief engagement (2–6 weeks); however, it may still underrepresent those who disengaged most severely or did not respond to recruitment efforts at all. Mental health conditions were self‐reported rather than clinically diagnosed; while appropriate given the study's focus on lived experience and perceptions of engagement, this means the severity and diagnostic specificity of participants' conditions cannot be confirmed. Retrospective self‐report may have introduced recall bias, particularly for participants reflecting on programmes attended up to 3 years prior, and social desirability bias may have shaped accounts in the context of an interviewer with professional experience in weight management. That professional background likely facilitated rapport and enabled nuanced interpretation of practitioner accounts but may also have oriented attentiveness toward service‐level barriers over individual‐level factors; ongoing reflexive journaling and supervisory challenge were used to surface and interrogate these assumptions throughout analysis. Single‐researcher coding, supported by a detailed coding manual, audit trail and supervisory review, inevitably carries interpretive subjectivity, though within the critical realist framework underpinning this study, researcher interpretation is treated as an analytic resource rather than a source of bias when made transparent and reflexively examined [57]. Given the nature and timescale of the current study, PPIE was primarily consultative; the advisory group was not recruited for intersectional diversity across ethnicity, socioeconomic position or gender identity, and contributors' own experiences of involvement were not formally evaluated. Finally, applying COM‐B/TDF to a constellation of interrelated behaviours rather than a single target behaviour introduced analytic complexity [36], though this reflected the ecological reality of T2WMS where eating, physical activity, emotion regulation and self‐regulation behaviours are addressed simultaneously.

### 4.3 Future Research

This study captured experiences at a single point in time; longitudinal qualitative research is needed to examine how the dynamic interplay between capability, opportunity and motivation evolves across the arc of programme engagement, including the critical transition from structured support to independent maintenance [19]. Building on the COM‐B/TDF mapping and BCW‐linked recommendations presented here, co‐produced intervention research should develop and test flexible, person‐centred service models that integrate co‐located psychological support, trauma‐informed facilitation, extended maintenance contacts and broader outcome measures. The predominantly White, female composition of this sample highlights the need for research that purposively recruits underrepresented groups including men, people from ethnically diverse communities, those taking psychotropic medication and individuals who have disengaged from services, to ensure that service redesign addresses rather than reproduces existing inequalities [53]. Future PPIE could move toward co‐production, involving contributors in theme consolidation and co‐developing recommendations [58].

## 5 Conclusion

This study demonstrates that engagement with health behaviour change among people with higher weight and symptoms of CMHPs seems to be shaped less by gaps in knowledge than by a dynamic, interconnected system of emotional, social and structural influences, within which motivation is itself embedded and continually reshaped, that current T2WMS provision is largely unequipped to address. The standardised, time‐limited model that treats weight loss as the primary measure of success, underpinning most programmes was, for many participants, experienced as misaligned with the emotional variability, relational needs and structural realities of this population. Sustained engagement may require a fundamental shift in how services are designed and evaluated, for example, embedding trauma‐sensitive facilitation, relational safety, flexible pacing and co‐located psychological support, while broadening outcome measures beyond weight to encompass wellbeing, functioning and quality of life. By systematically mapping lived experience to COM‐B, TDF and BCW intervention functions, this study provides an evidence‐based, theoretically grounded framework that commissioners, practitioners and policymakers can use to redesign T2WMS toward more inclusive, person‐centred and psychologically informed care.

## Author Contributions

**Veronika Rysinova:** conceptualisation, methodology, investigation, formal analysis, data curation, writing – original draft, writing – review and editing, project administration. **Sarah Brown:** supervision, methodology, validation, writing – review and editing. **Nicola Marie Stock:** supervision, methodology, validation, writing – review and editing.

## Ethics Statement

Ethical approval was obtained from the University of the West of England Faculty Research Ethics Committee (REF No: CHSS.24.05.201).

## Consent

All participants provided informed electronic consent prior to participation, including consent for anonymised quotations to be published.

## Conflicts of Interest

Veronika Rysinova was employed by Thrive Tribe, a provider of Tier 2 Weight Management services, during the completion of this study as part of her Professional Doctorate placement. Thrive Tribe funded the doctoral programme and supported participant recruitment. Beyond supporting recruitment, the funder had no role in study design, data collection, analysis, interpretation or the decision to submit for publication. Nicola Marie Stock and Sarah Brown declare no conflicts of interest.

## Supporting information


Supporting File 1

Supporting File 2

Supporting File 3

## Data Availability

Please contact the corresponding author for information about data availability. The data are not publicly available due to privacy and ethical restrictions, as they contain information that could compromise participant confidentiality.
