# Supplementary material for: Health Behaviour Change in Weight Management for People With Higher Weight and Symptoms of Common Mental Health Problems: A Qualitative Study
Source: Health Expect. 2026 Oct 25;29(5):e70906. doi: 10.1111/hex.70906 (PMC13696040; doi:10.1111/hex.70906)
Supplement: Supplementary file 1 [file HEX-29-e70906-s001.docx]

**Supplementary File 1**

**GRIPP2 Short Form Checklist: Reporting of Patient and Public Involvement and Engagement (PPIE)**

*Reported in line with the Guidance for Reporting Involvement of Patients and the Public (GRIPP2), short form (Staniszewska et al., 2017, BMJ).*

| **GRIPP2 domain** | **How addressed in this study** |
| --- | --- |
| **Aim** | To enhance the relevance and accessibility of the research by integrating experiential and professional insights into study design, recruitment materials, data collection tools, and interpretation of findings, and to monitor acceptability through participant feedback in order to continuously improve the research process. |
| **Methods** | A PPIE advisory group (n=4; two members with lived experience of higher weight and common mental health problems, two healthcare professionals delivering Tier 2 Weight Management Services) was consulted during study design, the ethics application, development of recruitment materials, and development of the interview topic guide, and during review of preliminary themes. Additionally, all study participants completed a brief post-interview survey to provide feedback on their experience and were invited to comment on preliminary findings. |
| **Study results** | Recruitment materials were revised to use clearer, more relatable, non-stigmatising language (e.g., ‘take part’ rather than ‘participate’; ‘people’ rather than ‘individuals’) and to remove ambiguity around eligibility for healthcare professionals. The interview topic guide was expanded to include emotional readiness and simplified language. Following early participant feedback, pre-interview communications were adjusted to better explain the nature of the questions and how to prepare. Thematic summaries were refined following participant review; for example, clarifying that ‘weight loss alone was rarely enough’ referred specifically to sustaining motivation and behaviour change, and more explicitly highlighting the perceived value of Tier 2 provision. Participant feedback confirmed that materials and thematic summaries were accessible and consistent with participants’ own experiences. For example, one participant expressed *“Thank you for inviting me to read the report, I think it is beautifully written and I know represents an accurate description of what we discussed. Thank you for taking the time to look into this when so many are not.”* |
| **Outcomes** | PPIE contributions shaped both the design and interpretive stages of the study: terminology, recruitment materials and the topic guide were adapted in response to advisory group input, and thematic language was refined following participant review of preliminary findings (respondent validation). One advisory group member also participated as an interview participant; their two contributions (advisory input and interview data) were treated as distinct and are reported separately in the manuscript. |
| **Critical perspective** | PPIE in this study was primarily consultative in nature: the advisory group and participants shaped materials, terminology and interpretation, but final analytic decisions remained with the researcher. This consultative approach, rather than a co-produced one, is acknowledged as a limitation of the study (see manuscript Discussion, 4.2). A formal evaluation of PPIE contributors’ own experience of the involvement process was not conducted, which is noted as an area for improvement in future work. Future research could move toward more co-productive approaches, sharing power in analytic decision-making and seeking wider demographic diversity within the advisory group. |

*Reference: Staniszewska S, Brett J, Simera I, et al. GRIPP2 reporting checklists: tools to improve reporting of patient and public involvement in research. BMJ. 2017;358:j3453. doi:10.1136/bmj.j3453*
