# Supplementary material for: Health Behaviour Change in Weight Management for People With Higher Weight and Symptoms of Common Mental Health Problems: A Qualitative Study
Source: Health Expect. 2026 Oct 25;29(5):e70906. doi: 10.1111/hex.70906 (PMC13696040; doi:10.1111/hex.70906)
Supplement: Supplementary file 2 [file HEX-29-e70906-s002.docx]

**Supplementary File 3**

**COREQ Checklist: Consolidated Criteria for Reporting Qualitative Research**

*Reported in line with Tong, Sainsbury & Craig (2007), International Journal for Quality in Health Care, 19(6):349–357.*

| **No.** | **Item** | **Description for this study** |
| --- | --- | --- |
| **Domain 1: Research team and reflexivity** | | |
| 1 | Interviewer/facilitator | The primary researcher (VR), a health psychology researcher, conducted all interviews. |
| 2 | Credentials | VR holds an MSc in Health Psychology and is a doctoral researcher. |
| 3 | Occupation | At the time of the study, VR was a doctoral student, researcher and mental health lead for a Tier 2 weight management provider, which funded the doctorate. |
| 4 | Gender | Female. |
| 5 | Experience and training | Extensive training in qualitative interviewing, thematic analysis, and psychological interventions. Prior experience delivering weight management and mental health support. |
| 6 | Relationship established | No prior relationship with lived-experience participants except one past client. The researcher introduced herself and the study purpose at recruitment. Most healthcare professional participants had an existing professional link with the researcher, as they were recruited via the same weight management provider. |
| 7 | Participant knowledge of interviewer | Participants were informed that the researcher was exploring experiences of weight management and mental health in Tier 2 services as part of a doctoral project. Both lived-experience participants and employees of the collaborating provider were made aware that participation was voluntary. |
| 8 | Interviewer characteristics | Reflexivity was maintained through a detailed journal, recognising the influence of the researcher’s professional background and values relating to non-stigmatising care. |
| **Domain 2: Study design** | | |
| 9 | Methodological orientation and theory | Codebook thematic analysis (Braun & Clarke), underpinned by the COM-B model and Theoretical Domains Framework (TDF). Situated within a critical realist ontology and contextualist interpretivist epistemology. |
| 10 | Sampling | Purposive sampling of adults with higher weight and common mental health problems who had attended Tier 2 programmes, alongside practitioners delivering such services. Sampling guided by the concept of information power. |
| 11 | Method of approach | Recruitment via programme providers, social media, and community outreach. |
| 12 | Sample size | 11 participants with lived experience; 11 healthcare professional participants. |
| 13 | Non-participation | A small number of individuals with lived experience initially expressed interest but did not respond to follow-up; reasons were not systematically captured. All approached healthcare professionals took part. |
| 14 | Setting of data collection | Online interviews conducted via Microsoft Teams. |
| 15 | Presence of non-participants | No one other than the participant and interviewer was present during interviews. |
| 16 | Description of sample | Adults aged 23–76, predominantly female and White British, with varied socioeconomic backgrounds. Practitioners included health coaches, lifestyle advisors, and specialist weight management staff. |
| 17 | Interview guide | Developed based on the COM-B model and TDF; shared with people with lived experience and health professionals in advance and adjusted for clarity and emotional accessibility (see Supplementary File 3). |
| 18 | Repeat interviews | None conducted. |
| 19 | Audio/visual recording | All interviews were audio-recorded with consent. |
| 20 | Field notes | Brief field notes were taken during and after interviews to capture immediate impressions. |
| 21 | Duration | Interviews ranged from 38 to 68 minutes (mean = 56 minutes). |
| 22 | Data saturation | Not numerically defined; sampling continued until thematic coverage was considered sufficient, guided by the concept of information power. |
| 23 | Transcripts returned | Transcripts were not returned to participants for review; participant validation instead took place via review of preliminary thematic findings, given the study’s focus on experiential themes rather than factual accuracy (see §2.6). |
| **Domain 3: Analysis and findings** | | |
| 24 | Number of data coders | Primary coding was conducted by VR; themes were developed and refined through collaborative discussion with supervisors, and a summary of preliminary findings was shared with all participants. |
| 25 | Description of the coding tree | A codebook comprising main codes, sub-codes, definitions and illustrative quotes was documented and maintained throughout analysis. |
| 26 | Derivation of themes | Themes were derived inductively from the data, then mapped deductively to COM-B and TDF domains to enhance explanatory depth. |
| 27 | Software | NVivo 14 was used for data management and coding. |
| 28 | Participant checking | Participants were invited to review preliminary findings (11 responses received). Minor adjustments were made in response. Analytic rigour was further supported through reflexivity, supervisory team discussion, and public involvement. |
| 29 | Quotations presented | Illustrative verbatim quotations are presented for each theme, attributed by pseudonym and participant group (lived experience or practitioner), with one contextually relevant characteristic included only where interpretively load-bearing. |
| 30 | Data and findings consistent | Clear links are shown between data extracts and interpretations throughout the results, to support analytic transparency. |
| 31 | Clarity of major themes | Five overarching themes and thirteen subthemes are clearly defined and illustrated with supporting data. |
| 32 | Clarity of minor themes | Minor patterns are discussed as nuances within the broader themes where relevant, rather than as standalone themes. |
