# Supplementary material for: Health Behaviour Change in Weight Management for People With Higher Weight and Symptoms of Common Mental Health Problems: A Qualitative Study
Source: Health Expect. 2026 Oct 25;29(5):e70906. doi: 10.1111/hex.70906 (PMC13696040; doi:10.1111/hex.70906)
Supplement: Supplementary file 3 [file HEX-29-e70906-s003.docx]

**Supplementary File 2**

**Interview Topic Guide**

*Barriers and facilitators to health behaviour change among adults with higher weight and common mental health problems in Tier 2 Weight Management Services: a qualitative study.*

*Developed with reference to the COM-B model (Michie, van Stralen & West, 2011) and the Theoretical Domains Framework (Cane, O’Connor & Michie, 2012). Reviewed and refined with input from the study’s patient and public involvement advisory group (see Supplementary File 1).*

Duration: approximately 45–60 minutes. Format: online via Microsoft Teams.

*Note: The interview schedule was applied flexibly and adjusted based on the flow of conversation and areas of interest or concern raised by the participant.*

**A. Semi-Structured Interview Schedule: Participants with Lived Experience**

**Introduction (interviewer script, summarised)**

The interviewer thanks the participant, introduces herself and her relationship to the study, and explains the purpose of the interview. Participants are reassured that no questions will ask about specific traumatic experiences or their weight directly, that they may skip any question or pause at any time, and that participation is voluntary. Confidentiality and its limits (risk of harm to self or others, abuse of a child or older adult, or disclosure of a crime) are explained, along with the right to withdraw data up to four weeks after interview. Consent to audio recording is reconfirmed before starting.

**Initial experiences**

- Tell me more about you and your journey so far.
- You took part in a weight management programme – what was your experience like?

**Capability – Psychological**

- What do you understand about the relationship between your weight, your mental health, and your overall wellbeing?
- Tell me about how things are now – are you making and keeping to healthy lifestyle changes? To what extent is this something you normally do?
- Is there anything making it more difficult to make those changes?
- How helpful has planning and monitoring things like eating habits, movement, stress and sleep been while accessing the programme, or since?

**Capability – Physical**

- What skills and abilities do you feel you need to make lifestyle changes that get you nearer to your health and weight goals?
- When you were accessing the programme, how did it go with gaining or using those skills and abilities? What went well? What was more challenging?

**Opportunity – Physical**

- Was there anything that made it more difficult at the time (for example, finding time or having enough money)?

**Opportunity – Social**

- Let’s talk about the people around you – family, friends. Were they helpful? Were they sometimes not helpful? How did they support you, or make things harder?
- In general, do you think of yourself as someone who maintains a healthy lifestyle?

**Motivation – Reflective**

- Since your weight management programme, how confident do you feel in your ability to make and sustain healthy lifestyle changes?
- What do you see as the main barriers to your confidence in making these changes? And the things that help?
- Are there aspects of your lifestyle you feel optimistic about changing for the better? Are there any you feel less optimistic about? Why?
- What benefits have you experienced since, or during, the programme? What benefits might there be in future?
- Are there any drawbacks or challenges to making these changes? How much do you think you’ll keep to them?
- What strategies have you put in place to help implement healthy lifestyle changes?

**Motivation – Automatic**

- What are the main reasons you’re likely to keep to these changes?
- How does making healthy lifestyle changes make you feel?

**Additional feedback**

- Is there anything else you would like to share about your experiences?

**Closing (interviewer script, summarised)**

The interviewer thanks the participant, confirms whether they would like a summary report of findings once available, restates the right to withdraw data within four weeks, reaffirms confidentiality, and confirms follow-up contact for a short post-interview feedback survey.

**B. Semi-Structured Interview Schedule: Healthcare Professional Participants**

**Introduction (interviewer script, summarised)**

As above: the interviewer introduces herself and the study, reassures the participant that questions will focus on their general professional experience rather than specific client cases, and reiterates confidentiality, its limits, voluntary participation and withdrawal rights. Consent to audio recording is reconfirmed before starting.

**Initial experiences**

- What is your experience of working in a weight management setting?
- You deliver a weight management programme – what has your experience been like?
- What typical initial experiences do you observe among clients with common mental health problems who take part in weight management programmes?

**Capability – Psychological**

- From your professional standpoint, what is the relationship between weight, mental health and overall wellbeing?
- To what extent is making healthy lifestyle choices something your clients normally do?
- What contributes to difficulties in remembering or prioritising these changes among clients?
- How do monitoring, planning or habit-forming strategies help or hinder clients in weight management programmes?

**Capability – Physical**

- What skills and abilities do you believe are important for clients to successfully make lifestyle changes in weight management programmes?
- What challenges or successes do clients with common mental health problems encounter when developing or using these skills within the programme?

**Opportunity – Physical**

- What external factors tend to help or hinder clients with common mental health problems in making health-promoting lifestyle changes (for example, finding time or having enough money)?

**Opportunity – Social**

- To what extent do social influences (e.g., family or friends) affect clients’ ability to make lifestyle changes within the programme?
- Do you typically see clients maintaining a healthy lifestyle outside the programme?

**Motivation – Reflective**

- How confident do clients with common mental health problems seem in their ability to make and sustain healthy lifestyle changes within the programme?
- What are the main barriers, and the main boosters, to clients’ confidence in making these changes?
- Are there aspects of clients’ lifestyles you observe optimism about changing? Any where clients express less optimism? What contributes to this?
- What benefits do clients experience from adopting a healthier lifestyle within the programme? What drawbacks or challenges do they perceive?
- To what extent do clients express intentions to maintain lifestyle changes? What strategies have they shared for doing so?

**Motivation – Automatic**

- What reasons or incentives have you observed to be most effective in motivating clients to maintain a healthy lifestyle?
- How do clients typically describe the emotional impact of making healthy lifestyle changes within the programme?

**Additional feedback**

- Is there anything else you would like to share?

**Closing (interviewer script, summarised)**

The interviewer thanks the participant for their time and professional insight, and confirms follow-up procedures as above.
